# Atrial Fibrillation After Coronary Artery Bypass Grafting and Its Relationship with Hospital Complications in São Paulo State

**DOI:** 10.21470/1678-9741-2023-0270

**Published:** 2024-05-13

**Authors:** Adnaldo da Silveira Maia, Dayara Hoffmann Mayer, Renê Augusto Gonçalves e Silva, Andresa Fernandes Pérego, Pedro Esteban Ulloa Alvarado, Oscar Harold Torrico Lizarraga, Mercy Adriana Herrera Arcos, Matheus da Silveira Maia, Magaly Arrais dos Santos, Omar Asdrubal Vilca Mejia

**Affiliations:** 1 Department of Cardiovascular Surgery, Instituto Dante Pazzanese de Cardiologia (IDPC), São Paulo, São Paulo, Brazil; 2 Department of Cardiovascular Surgery, Hospital do Coração (HCor), São Paulo, São Paulo, Brazil; 3 Department of Cardiovascular Surgery, Hospital Regional do Baixo Amazonas (HRBA), Santarém, Pará, Brazil; 4 Department of Surgery, Faculdade de, Universidade do Estado do Pará (UEPA), Belém, Pará, Brazil; 5 Instituto do Coração (InCor), Faculdade de Medicina, Universidade de São Paulo (USP), São Paulo, São Paulo, Brazil

**Keywords:** Database, Coronary Artery Bypass, Atrial Fibrillation, Mortality

## Abstract

**Introduction:**

Atrial fibrillation is the main complication in the postoperative period of cardiovascular surgery. Its genesis is multifactorial, so its rapid identification to mitigate the associated risks is essential.

**Objective:**

To evaluate the incidence of atrial fibrillation in patients undergoing coronary artery bypass grafting (CABG) and its relationship with other complications in our setting.

**Methods:**

This is a multicenter, observational study involving patients undergoing isolated CABG between 2017 and 2019 with data from the Registro Paulista de Cirurgia Cardiovascular (or REPLICCAR II). Variables were prospectively collected in REDCap following the definitions given by version 2.73 of the Society of Thoracic Surgeons Adult Cardiac Surgery Database. Data were collected with prior authorization from the local ethics committee and analyses performed in R software.

**Results:**

A total of 3,803 patients were included, of these 605 had postoperative atrial fibrillation (POAF). In order to adjust the groups, propensity score matching was used. Such analyses resulted in 605 patients in each group (without POAF vs. with POAF). Among patients with POAF, the mean age was 67.56 years, with a prevalence of males (73.6%, 445 patients). Patients belonging to the group with POAF had a mortality rate of 9.26% (P=0.007), longer ventilation time (P<0.001), pneumonia (P<0.001), and sepsis (P<0.001). In multiple analysis, acute renal dysfunction (P=0.032) and longer intensive care unit stay (P<0,001) were associated with the presence of POAF.

**Conclusion:**

POAF in CABG is associated with longer intensive care unit and hospital stay, as well as renal dysfunction, pneumonia, and in-hospital mortality.

## INTRODUCTION

**Table:** 

Abbreviations, Acronyms & Symbols				
AF	= Atrial fibrillation		EuroSCORE	= European System for Cardiac Operative Risk Evaluation
BMI	= Body mass index		Hb	= Hemoglobin
CABG	= Coronary artery bypass grafting		Ht	= Hematocrit
CCS	= Canadian Cardiovascular Society		IABP	= Intra-aortic balloon pump
CHA₂DS₂-VASC	= Congestive heart failure, Hypertension, Age ≥ 75 years (doubled), Diabetes mellitus, prior Stroke or TIA or thromboembolism (doubled), Vascular disease, Age 65 to 74 years, Sex category		ICU NYHA OR POAF	= Intensive care unit = New York Heart Association = Odds ratio = Postoperative atrial fibrillation
CI	= Confidence interval		PP	= Posterior pericardiotomy
CPB	= Cardiopulmonary bypass		RBC	= Red blood cell
ECG	= Electrocardiogram		REPLICCAR	= Registro Paulista de Cirurgia Cardiovascular

Coronary artery disease is the leading cause of death in developing countries. Its genesis is multifactorial, involving factors such as lifestyle and genetic predisposition. Coronary artery bypass grafting (CABG) has been well established since the 1960s in the management of such patients^[1]^.

Atrial fibrillation (AF) is the main arrhythmia present in the postoperative period of cardiovascular surgery. It is estimated to have an incidence of 15-40%, especially in combined procedures, *i.e.*, those which include CABG and valve surgery. Therefore, in patients undergoing mitral valve surgery, 64% may present postoperative atrial fibrillation (POAF)^[2,3]^.

Among the cases, 90% occur in the first four postoperative days, with recurrence of 40% in 24 hours with peak incidence on the second day. However, it is estimated that 80% of patients return to sinus rhythm^[3]^. It is emphasized that the preoperative presence of AF is associated with an increase in adverse events and mortality^[4,5]^.

Among the risk factors, age stands out as the main one involved. For every 10-year increase after the age of 50, there is an increase of approximately 13% in the risk of POAF. Other factors include hypertension, diabetes mellitus, obesity, and chronic obstructive pulmonary disease^[3]^. Some parameters obtained by echocardiography help to predict the presence of AF. In a study conducted by Lu et al.^[6]^ in a prospective cohort involving 126 patients undergoing CABG, the left atrial diameter and pulmonary artery systolic pressure obtained a significantly statistical association. Similar results were obtained by Onk et al.^[7]^ who evaluated 256 patients undergoing CABG.

Kang et al.^[8]^ identified findings in agreement with the aforementioned authors in patients undergoing valve surgery, corroborating the thesis that the left atrial diameter is an important factor in the basis of AF.

The mechanisms involved in the genesis of this arrhythmia are still uncertain; however, it is known that the surgical inflammatory process and the excessive production of catecholamines are associated. Furthermore, neurohormonal activation increases the susceptibility of patients to AF through sympathetic and parasympathetic activity that alters the atrial refractory period, serving as a substrate for the origin of this arrhythmia^[9]^.

Several papers discuss the use of certain drugs in order to prevent the incidence of AF, including beta blockers, amiodarone, magnesium, and statins with still conflicting results^[10-12]^.

Despite the different risk factors and multifactorial pathophysiology, the presence of POAF is associated with increased complications in this critical period. These include stroke, intracardiac thrombus formation, hypotension, acute pulmonary edema, and acute renal injury. Other correlated complications include longer hospital stay as well as longer length of stay in the intensive care unit (ICU)^[13-15]^.

Thus, the aim of this study was to analyze the relationship of AF in the postoperative period of isolated CABG with hospital complications that may be associated with this arrhythmia. The analysis was performed using the Registro Paulista de Cirurgia Cardiovascular (REPLICCAR II) (São Paulo, Brazil), a multicenter database including 3,803 patients.

## METHODS

### Study Population

This is a multicenter, prospective, observational study. Only patients undergoing primary isolated CABG in the period between 2017-2019 belonging to the REPLICCAR II were included. Variables were prospectively collected in REDCap following the definitions given by version 2.73 of the Society of Thoracic Surgeons Adult Cardiac Surgery Database. The use of the database was approved by the Research Ethics Committee of the Instituto do Coração, Hospital das Clínicas, Faculdade de Medicina, Universidade de São Paulo. Informed consent was obtained from each of the participants. Demographic, preoperative, intraoperative, and in-hospital evolution data of the patients were collected and recorded.

### Definitions and Clinical-Surgical Management

Patients who had any episodes of AF in the in-hospital postoperative period were included. POAF was defined as any evidence of AF observed by electrocardiogram (ECG) or continuous monitoring for a minimum of 30 minutes.

The clinical management of the patients consisted in maintaining the use of beta-blockers until the day of the procedure. For patients undergoing cardiopulmonary bypass (CPB), moderate hypothermia of 32 to 34°C was used. Myocardial protection was performed by intermittent clamping, crystalloid, or blood cardioplegia according to the surgeon’s preference. In the postoperative period, patients were continuously monitored with daily ECG and maintenance of beta-blockers, except for patients with contraindication. In the presence of AF for > 48 hours, anticoagulation was initiated.

After hospital discharge, the patients were referred for outpatient follow-up with clinical management as recommended by national and international guidelines.

### Statistical Analysis

The analysis was performed using the R software version 4.2.0. As the continuous variables did not follow the normal distribution (Anderson-Darling test), the nonparametric Mann-Whitney and Brunner-Munzel tests were used to compare two groups for homogeneous and heterogeneous variables (Bartlett’s test), respectively. For categorical variables, the Fisher’s exact test or the Chi-square test was used.

In order to adjust the groups, propensity score matching was used, using the variables chronic/acute renal failure, New York Heart Association classification, diabetes mellitus, peripheral arterial disease, former smoker/smoker, sex, acute myocardial infarction, need for intra-aortic balloon, surgical status, and intraoperative blood product transfusion. There was no statistical difference between the variables selected for the adjustment between the groups in order to avoid any bias in the analysis. A multivariate logistic regression model was also used to identify factors associated with POAF. The significance level adopted was 0.05, with a 95% confidence interval.

## RESULTS

We included 3,803 patients present in the REPLICCAR II database and they were divided into two groups: with and without POAF. We observed in the group that presented POAF male prevalence 73.6% (*P*=0.92) and statistical difference in variables such as functional class (*P*<0.001), need for intra-aortic balloon (*P*<0.001), mediastinitis (*P*<0.001), renal dysfunction (*P*<0.001), and mortality (*P*<0.001). Other variables are shown in Table 1.

**Table 1:** Demographic and clinical characteristics.

Variable	Non-POAF (n = 3198)	POAF (n = 605)	*P*-value
Age	62.47 ± 9.1	67.56 ± 8.3	< 0.001
Sex			
Male	2348 (73.4%)	445 (73.6%)	0.92
Systemic arterial hypertension	2827 (88.4%)	545 (90.08%)	0.263
Diabetes mellitus	1555 (48.62%)	311 (51.4%)	0.215
Dyslipidemia	1967 (61.51%)	378 (62.48%)	0.682
Peripheral arterial disease	226 (7.08%)	46 (7.6%)	0.667
Current smoker	498 (17.63%)	71 (14%)	0.047
Former smoker	901 (31.89%)	188 (37.08%)	0.024
EuroSCORE	1.75 ± 1.59	2.45 ± 2.83	< 0.001
Ejection fraction	57.3 ± 12.38	55.4 ± 12.85	0.002
Functional class			
NYHA I	1484 (47.34%)	271 (46.56%)	0.001
NYHA II	505 (16.11%)	77 (13.23%)	
NYHA III	291 (9.28%)	83 (14.26%)	
NYHA IV	101 (3.22%)	26 (4.47%)	
CCS class			
CCS I	362 (11.6%)	66 (11.44%)	0.978
CCS II	742 (23.77%)	135 (23.4%)	
CCS III	511 (16.37%)	98 (16.98%)	
CCS IV	310 (9.93%)	61 (10.57%)	
Preoperative creatinine clearance	76.03 ± 29.22	67.49 ± 27.43	< 0.001
Preoperative Hb	13.52 ± 1.82	13.33 ± 1.83	0.033
Preoperative Ht	40.01 ± 4.87	39.40 ± 5.48	0.032
Surgery status			
Elective	2105 (65.9%)	368 (60.93%)	0.039
Emergency	13 (0.41%)	4 (0.66%)	
Urgency	1076 (33.69%)	232 (38.41%)	
Need for IABP	106 (3.31%)	56 (9.26%)	< 0.001
Postoperative stroke	35 (1.09%)	21 (3.47%)	< 0.001
Mediastinitis	107 (3.35%)	41 (6.78%)	< 0.001
Postoperative acute renal dysfunction	150 (4.69%)	123 (20.33%)	< 0.001
Readmission to ICU in 30 days	119 (4.27%)	21 (4.69%)	0.707
Reintubation	64 (2.01%)	59 (9.75%)	< 0.001
Sepsis	87 (2.96%)	70 (11.57%)	< 0.001
Mortality	63 (1.97%)	56 (9.26%)	< 0.001

CCS=Canadian Cardiovascular Society; EuroSCORE=European System for Cardiac Operative Risk Evaluation; Hb=hemoglobin; Ht=hematocrit; IABP=intra-aortic balloon pump; ICU=intensive care unit; NYHA=New York Heart Association; POAF=postoperative atrial fibrillation

We performed a propensity score matching, by selecting variables from the database, in order to match the groups with and without POAF (605 patients in each group). We could observe a higher prevalence of males (*P*=0.793), without statistical difference regarding comorbidities, including hypertension, dyslipidemia, peripheral arterial disease, and smoking. There was no difference between the groups when analyzing the methods of myocardial protection, blood cardioplegia *vs.* crystalloid *vs.* intermittent clamping (*P*=0.85). However, factors such as need for dialysis in the postoperative period, prolonged length of stay (24 hours), pneumonia, readmission to ICU, and sepsis were associated with AF (*P*<0.001). It is worth noting that there was statistical difference when we analyzed the outcome mortality alone (Table 2).

**Table 2:** Propensity score matching.

Variable	No AF (n = 605)	With AF (n = 605)	*P*-value
Age	67.51 ± 8.05	67.56 ± 8.30	0.931
Sex			
Male	450 (74.38%)	445 (73.68%)	0.793
Systemic arterial hypertension	537 (88.76%)	545 (90,08%)	0.513
Diabetes mellitus	301 (49.75%)	311 (51.4%)	0.605
Dyslipidemia	356 (58.84%)	378 (62.48%)	0.217
Peripheral arterial disease	54 (8.94%)	46 (7.6%)	0.406
Smoker	62 (12.33%)	71 (14%)	0.457
Former smoker	191 (37.97%)	188 (37.08%)	0.795
EuroSCORE	2.33 ± 2.19	2.45 ± 2.83	0.676
Ejection fraction	56.46 ± 13.02	55.43 ± 12.85	0.138
Functional class			
NYHA I	275 (47.5%)	271 (46.56%)	0.940
NYHA II	81 (13.99%)	77 (13.23%)	
NYHA III	73 (12.61%)	83 (14.26%)	
NYHA IV	25 (4.32%)	26 (4.47%)	
Preoperative creatinine clearance	65.85 ± 27.13	67.49 ± 27.43	0.300
Postoperative creatinine	1.65 ± 1.30	2.04 ± 1.92	0.001
Postoperative dialysis need	13 (2.54%)	43 (8.11%)	< 0.001
Reoperation	8 (1.46%)	21 (3.47%)	0.037
Prolonged postoperative length of stay (> 14 days)	62 (10.73%)	144 (25.26%)	< 0.001
Prolonged ventilation time (> 24 h)	38 (6.31%)	77 (12.77%)	< 0.001
Postoperative stroke	4 (0.66%)	21 (3.47%)	0.001
Mediastinitis	27 (4.46%)	41 (6.78%)	0.104
Pneumonia	21 (3.83%)	54 (8.93%)	< 0.001
ICU readmission	19 (3.14%)	93 (15.37%)	< 0.001
Reintubation	24 (3.98%)	59 (9.75%)	< 0.001
Sepsis	30 (5.47%)	70 (11.57%)	< 0.001
Mortality	31 (5.12%)	56 (9.26%)	0.007

AF=atrial fibrillation; EuroSCORE=European System for Cardiac Operative Risk Evaluation; ICU=intensive care unit; NYHA=New York Heart Association

Logistic regression was performed with the variable “postoperative atrial fibrillation” present in the REPLICCAR II database. Based on variables with statistical significance, a multiple model was built (Table 3).

**Table 3:** Univariate and multivariate logistic regression for postoperative atrial fibrillation.

Variable	Univariate analysis OR (95% CI)	*P*-value	Multivariate analysis OR (95% CI)	*P*-value
Age	1.001 (0.987-1.015)	0.913		
Sex	1.037 (0.802-1.341)	0.780		
BMI	1.00 (0.990-1.001)	0.978		
Systemic arterial hypertension	1.15 (0.797-1.66)	0.455		
Dyslipidemia	1.165 (0.925-1.467)	0.196		
Peripheral obstructive artery disease	0.838 (0.556-1.264)	0.399		
Diabetes mellitus	1.068 (0.853-1.339)	0.565		
Prior acute myocardial infarction	1.101 (0.878-1.380)	0.406		
EuroSCORE	1.018 (0.973-1.066)	0.438		
Ejection fraction	0.994 (0.985-1.004)	0.234		
Functional class				
NYHA II	0.965 (0.677-1.375)	0.842		
NYHA III	1.154 (0.808-1.648)	0.432		
NYHA IV	1.055 (0.594-1.874)	0.854		
Pulmonary artery systolic pressure (mmHg)	0.986 (0.965-1.007)	0.199		
Acute renal failure	2.89 (2.03-4.12)	< 0.001	1.72 (1.04-2.83)	0.032
Postoperative dialysis need	3.38 (1.80-6.38)	< 0.001	1.12 (0.474-2.66)	0.790
CPB time (min)	1.002 (0.998-1.006)	0.400		
Length of stay in ICU (hours)	1.005 (1.003-1.007)	< 0.001	1.005 (1.003-1.007)	< 0.001
Mechanical ventilation time	1.004 (1.001-1.006)	0.002		
Postoperative RBC transfusion	1.102 (0.960-1.265)	0.168	1.27 (0.945-1.71)	0.112
Reoperation	2.42 (1.06-5.52)	0.035	1.37 (0.535-3.54)	0.506
Need for IABP	1.26 (0.843-1.91)	0.254		

BMI=body mass index; CI=confidence interval; CPB=cardiopulmonary bypass; EuroSCORE=European System for Cardiac Operative Risk Evaluation; IABP=intra-aortic balloon pump; ICU=intensive care unit; NYHA=New York Heart Association; OR=odds ratio; RBC=red blood cell

From the multiple regression analysis, we can infer:

- Patients who evolved with postoperative acute renal failure have 1.72 more chance of presenting POAF compared to patients who did not present this outcome.
- Patients who had prolonged length of stay in ICU have 1.005 more chance of presenting POAF.

## DISCUSSION

AF is described as the most frequent arrhythmia in the postoperative period of cardiac surgery, and its incidence after isolated CABG is between 30 and 40%^[1,3,13]^. The occurrence of such arrhythmia implies a worse prognosis due to prolonged hospitalization and higher associated morbidity, generating adverse health effects. Thus, the need to identify associated factors arises to provide the implementation of measures in an attempt to reduce its occurrence^[1,3]^.

The number of publications on the subject has grown dramatically in recent decades, increasing from < 100 to 600 articles in 2020. Several mechanisms are involved in the genesis of this complication, including atrial structural changes, presence of pericardial effusion, myocardial ischemia, and autonomic neuromodulation^[16]^.

Geovanini et al.^[17]^ in a Brazilian observational study, which included 66 patients in the postoperative period of elective cardiac surgery (CABG and/or valve surgery) with mean age of 62 years, correlated two main risk factors regarding the emergence of POAF, being the most prevalent advanced age (mainly age > 65 years) and left atrial size. Of the 3,803 patients included in our database, a higher incidence of POAF was also observed in those with older age (67.56 ± 8.3) when compared to those who did not develop POAF.

Regarding the demographic characteristics, Musa et al.^[2]^, in their retrospective study which included 637 patients undergoing isolated CABG, showed a predominance of males in the development of POAF, as it is also evidenced in this study (79.2% *vs.* 73.6%). In agreement with the study of these authors, despite the high prevalence of the main comorbidities such as hypertension, diabetes mellitus, and hypercholesterolemia, in both groups (with POAF and without POAF) such conditions did not demonstrate statistically significant difference for the emergence of postoperative arrhythmia. No relationship with smoking was demonstrated either. On the other hand, patients with chronic kidney disease diagnosed preoperatively had a higher incidence of POAF in the cited study, and in our analysis, there was also a statistically significant correlation between lower preoperative creatinine clearance and occurrence of AF. Mangi et al.^[1]^, in their prospective study that involved 163 patients submitted to isolated CABG, observed an incidence of POAF in 25.8%. In our study, we had an even lower number of POAF (15.9%), even with a larger sample size (3,803 *vs.* 163). However, within the expected values in the postoperative setting (15-40%).

It was evidenced in the present study an association of POAF with longer ICU bed length of stay and increased mechanical ventilation time. Infectious complications were also seen, such as sepsis, pneumonia, besides the need for reintubation, readmission to ICU, and postoperative stroke, which among all the complications of POAF, remains the most feared due to the high morbidity associated^[1,14]^.

The study by Musa et al.^[2]^ also showed a six-fold increase in the incidence of stroke in the POAF group, but without reaching statistical significance. In our study, we obtained similar results - although the analysis by propensity score matching showed an increase in the stroke rate in the POAF group with a significant *P*-value, when univariate logistic regression was performed, the same result was not obtained.

Mechanical ventilation time and the need for reoperation showed relevance in the emergence of POAF in univariate analysis, but statistical significance was lost after adjustment for multivariate analysis. The results of the multiple regression analyses showed statistical significance in relating POAF to acute renal failure and prolonged ICU stay. NG et al.^[15]^ had already demonstrated a two times higher risk of patients with acute renal failure developing POAF in the context of postoperative heart surgery, which can be explained by the low clearance of pro-inflammatory cytokines generated by surgical trauma and use of CPB, besides the increase of oxidative stress capable of altering the functioning of cellular ion channels, interfering directly in the electrical stability of the cell membrane and in the conduction of atrial electrical impulses, leading to the emergence of arrhythmias.

Importantly, prior pathological history also has a direct implication with in-hospital mortality in patients presenting with POAF, as demonstrated in a cohort study that included 1,390 patients undergoing CABG, with the most associated being a personal history of stroke, acute myocardial infarction, and percutaneous coronary intervention^[17]^.

Thus, some studies try to predict POAF incidence, using some scores as POAF and CHA2DS2-VASC (which stands for Congestive heart failure, Hypertension, Age ≥ 75 years [doubled], Diabetes mellitus, prior Stroke or TIA or thromboembolism [doubled], Vascular disease, Age 65 to 74 years, Sex category)^[18]^. Chua et al.^[19]^, in an analysis of 277 patients undergoing cardiac surgery, demonstrated after multivariate regression the relationship between high scores and POAF. Similar results were obtained by Uysal et al.^[20]^ when evaluated 370 patients undergoing CABG, where the CHA2DS2-VASC score was able to predict POAF, becoming a tool in the detection of patients at higher risk.

In the scope of prevention of AF after CABG, the correction of hydroelectrolytic disorders associated with the preoperative and/or postoperative administration of antiarrhythmic medications are effective. Even the use of beta-blockers preoperatively stands out with a high degree of recommendation according to the main guidelines (American College of Cardiology/American Heart Association/European Society of Cardiology) and followed in the protocol of this study^[7,9,11,13]^.

Although many patients return to sinus rhythm spontaneously, when this does not occur, the treatment consists mainly of administering drugs capable of controlling the heart rate. Anticoagulation should be indicated if the AF rhythm persists > 48 hours, depending on the CHA₂DS₂-VASC^[13]^. The national AF guidelines also points out percutaneous occlusion devices of the left atrial appendage in addition to surgical approaches such as the Cox-Maze procedure and hybrid treatments^[21]^.

Recent studies show that the left posterior pericardiotomy (PP) technique is associated with a reduction in POAF. A meta-analysis of 4,467 patients in 25 randomized clinical trials of patients undergoing cardiac surgery comparing the PP group with the control group showed a significant reduction in POAF in patients assigned to the PP group (odds ratio 0.49, [0.38-0.61])^[22,23]^. In addition, the perioperative optimization of patients can contribute to the reduction of POAF with Enhanced Recovery After Surgery (or ERAS), which include multimodal anesthesia, rapid extubation, early drain removal after bleeding reduction and ultrasound confirmation, quick ambulation, and follow-up by a multidisciplinary team. In Brazil, Mejia et al.^[24]^ demonstrated the effectiveness of these measures.

Regarding the outcome of stroke, some studies have shown that the surgical technique without CPB would have a positive effect in reducing postoperative neurological complications. This observation, however, was not part of the scope of the present analysis^[14]^.

### Limitations

The present study has some limitations. We can list it as having been the result of secondary analysis of the REPLICCAR II database. In addition, we do not have follow-up information on these patients beyond the information present in this study. Other factors associated with AF such as quality of life or longer follow-up of patients should be evaluated in order to better understand the long-term impact of this complication.

## CONCLUSION

In the present study, it was demonstrated a close relationship between POAF and several hospital complications, mainly related to acute renal failure and longer ICU stay. Strategies to reduce the incidence of this complication after cardiovascular surgery in the state of São Paulo, such as establishing institutional enhanced recovery protocols and expanding the use of techniques such as left PP, could be alternatives and contribute to improving surgical outcomes and patient care.

### Study Association

Presented at The 49^th^ Brazilian Congress of Cardiovascular Surgery, Maceió, Brazil, May 26-27, 2023.
